# Steps towards a phylogeny of the pill millipedes: non-monophyly of the family Protoglomeridae, with an integrative redescription of *Eupeyerimhoffia archimedis* (Diplopoda, Glomerida)

**DOI:** 10.3897/zookeys.510.8675

**Published:** 2015-06-30

**Authors:** Jan Philip Oeyen, Thomas Wesener

**Affiliations:** 1 Zoologisches Forschungsmuseum Alexander Koenig, Leibniz Institute for Animal Biodiversity, Center for Taxonomy and Evolutionary Research (Section Myriapoda), Adenauerallee 160, D-53113 Bonn, Germany

**Keywords:** COI, Glomerida, integrative, taxonomy, redescription, Sicily

## Abstract

*Eupeyerimhoffia archimedis* (Strasser, 1965) is redescribed based on several specimens collected at a number of sites close to the type locality on Sicily, Italy. Scanning electron microscopy is used to illustrate several unusual morphological characters for a member of the Glomerida for the first time. A fragment of the mitochondrial COI gene (668bp) is sequenced for the first time in *Eupeyerimhoffia* to provide a species-specific barcode and to gain first insights into the genetic distances between the genera in the widespread family Protoglomeridae. The novel sequences are compared to representatives of all other genera of the family: *Protoglomeris vasconica* (Brölemann, 1897) from northern Spain, the dwarfed *Glomerellina laurae* Silvestri, 1908 from Italy and *Glomeroides primus* (Silvestri, 1929) from western North America. The addition of COI sequences from the two other families of the Glomerida renders the family Protoglomeridae paraphyletic with *Glomeroides primus* being more closely related to *Glomeridella minima* (Latzel, 1884) than to the other genera in the family. The large genetic distances (13.2–16.8%) between *Eupeyerimhoffia* and the other genera in the order, as well as its unusual morphological characters, including unique morphological adaptations to roll into a ball, are probably an indication of the old age of the group.

## Introduction

The pill millipedes of the order Glomerida comprise about 290 species in 34 genera (Mauriès 2005, Golovatch et al. 2010, Wesener 2010, 2012) and exhibit a Holarctic distribution, with species found in North America, Europe and North Africa, and Asia with the exception of India south of the Himalayas (Shelley and Golovatch 2011). The Glomerida are currently divided into three families (Mauriès 1971, 2005), the two species-poor families Glomeridellidae and Protoglomeridae, and the family Glomeridae, which contains the majority of species and genera (~240 species in 27 genera) (Mauriès 2005, Wesener 2012).

While the two genera of the Glomeridellidae are Mediterranean, the four genera and 20 species of the Protoglomeridae show a disjunct distribution, partly European, in Spain, the eastern Mediterranean, Algeria and Sicily, and partly in the New World from Guatemala to California (Mauriès 2005).

Here we redescribe the little-known species *Eupeyerimhoffia archimedis* (Strasser, 1965), and describe the male telopods for the first time. Additionally, we illustrate several unusual (and potentially apomorphic) morphological characters of a member of the family Protoglomeridae for the first time using scanning electron microscopy. To complete our integrative approach, we also analyze the genetic distances between the four genera of the family using the common barcoding fragment, COI.

## Material and methods

**Figure 1.**
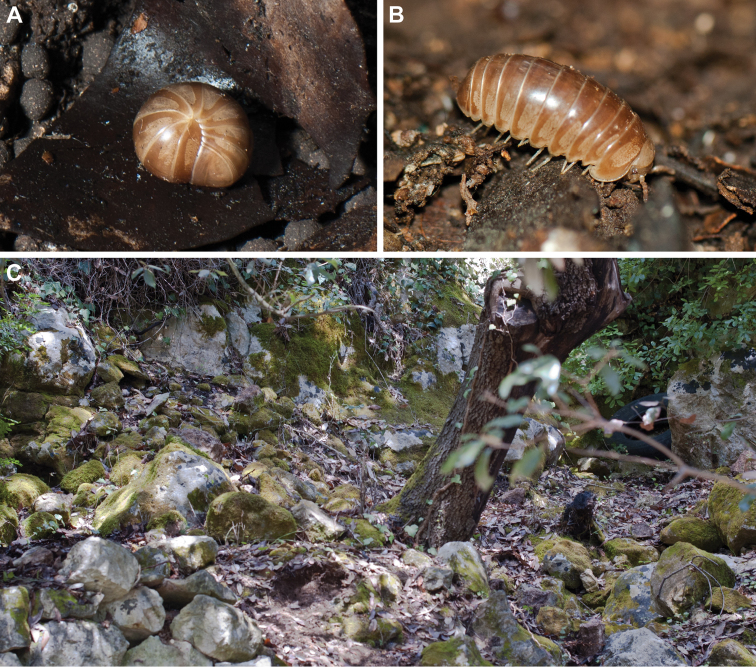
*Eupeyerimhoffia archimedis* (Strasser, 1965) female in situ and habitat. **A** *Eupeyerimhoffia archimedis* female rolled up, in situ **B** *Eupeyerimhoffia archimedis* female in situ **C** Habitat of *Eupeyerimhoffia archimedis*, close to the type locality Ferla.

Samples of *Eupeyerimhoffia archimedis* were collected by hand in July 2013. A single male and several females were collected close to the type locality (Ferla; Fig. 1C) and further samples were collected at a new locality (East of Palazzolo Acreide, Sicily). Exact coordinates are provided in Table 1. All samples were conserved in 98% EtOH for further analyses and deposited in the collection of the Zoological Research Museum Alexander Koenig (ZFMK, Bonn, Germany).

**Table 1.** Sample information with voucher numbers (ZFMK = Zoological Research Museum Alexander Koenig, Bonn, Germany. ZSM = Bavarian State Collection of Zoology. NHMC = Natural History Museum of Crete), GenBank accession numbers (Acc.#) and locality information. Samples where sequences were downloaded from GenBank are marked with an asterisk.

Species	Specimen Voucher	Acc. #	Locality
*Glomeris marginata (Villers, 1789)	ZFMK MYR0009	FJ409909	Germany, Nordrhein-Westfalen, Bonn, Venusberg, coll. T. Wesener, IX.2007
*Glomeridella minima (Latzel, 1884)	ZFMK MYR0003	JQ074181	Slovenia, Lower Sava, Brežice, Prilipe, dry creek valley, 45.8773°N, 15.6246°E, 150 m, coll. H. Reip, 17.x.2009.
*Geoglomeris subterranea Verhoeff, 1908	BC ZSM MYR 00370	JQ350441	Switzerland, Aargau
*Trachysphaera sp.	ZFMK MYR0006	JQ074180	Italy, Piemonte, Biella, NW Sanctuary of Oropa, Fagus forest with stones, 45.62947°N, 7.98168°E, 1200 m, coll. T. Wesener, 14.iv.2011
*Glomeroides primus (Silvestri, 1929)	ZFMK MYR0004	JQ074182	U.S.A., California, Mendocino County, between Fort Bragg and Whiskey Springs, 39.3976°N, 123.6946°W, 35 m, coll E. Garcia, C. Richart & A. Schönhofer, 29.iii.2011.
*Onychoglomeris tyrolensis* (Latzel, 1884)	ZFMK MYR1276	KP205571	Italy, Trentino-Südtirol, Prov. Trient, Madonna di Campiglio, Beech forest, 46.2209528°N, 010.8296250°E, 1553 m, coll. T. Wesener, 04.x.2012.
*Protoglomeris vasconica* (Brölemann, 1897)	ZFMK MYR0934	KP205572	Spain, Galicia, Ribadeo, Trabada, deep and moist creek valley with deciduous forest, 43.4295°N, 7.2290°E, coll. H. Reip, 29.vii.2012.
*Glomerellina laurae* Silvestri, 1908	ZFMK MYR2260	KP205573	Europe, Greece, Rhodos, Kapi - Profitis Ilias, coll. NHMC, 01.i.2000.
*Eupeyerimhoffia archimedis* (Strasser, 1965) 1	ZFMK MYR1876	KP205574	Italy Sicily, Province Syracuse, South of Ferla, Southern slope, deciduous forest, 37.1151333°N, 014.9403667°E, coll. J.P. Oeyen & P. Erkeling, 10.vii.2013.
*Eupeyerimhoffia archimedis* (Strasser, 1965) 2	ZFMK MYR1965	KP205575	Italy, Sicily, Province Syracuse, East of Palazzolo Acreide, Ravine, deciduous forest, 37.0997667°N, 015.0232000°E, coll. J.P. Oeyen & P. Erkeling, 13.vii.2013.

### Morphological analysis

A female and the single male from the type locality were dissected under an Olympus SZX12 stereomicroscope with Dumont 5 Inox B forceps. Samples were dehydrated in 100% EtOH for 12 hrs, mounted on aluminum stubs, dried for 12 hrs at 45 °C and sputter coated with 50 nm of pure gold in a Hummer VI sputtering system (Anatech LTD, USA). Samples were observed with a Hitachi S-2460N SEM (Hitachi LTD, Japan) and digital images were captured using DISS5 (point electronic GmbH, Germany).

### Molecular analysis

Muscle tissue was removed from specimens of *Onychoglomeris tyrolensis* (Latzel, 1884), *Protoglomeris vasconica* (Brölemann, 1897), *Glomerellina laurae* Silvestri, 1908, and *Eupeyerimhoffia archimedis* (Strasser, 1965). Sequences of *Glomeroides primus* (Silvestri, 1929) were downloaded from GenBank. Additionally, sequences from GenBank of *Glomeridella minima* (Latzel, 1884), a member of the basal family Glomeridellidae, as well as of *Glomeris marginata* (Villers, 1789), *Geoglomeris subterranea* Verhoeff, 1908 and *Trachysphaera* sp. from the family Glomeridae (Table 1) were also downloaded. Specimens from which DNA was extracted were stored as vouchers at the ZFMK. Accession numbers, locality data and voucher information for all samples included in the study are displayed in Table 1.

Total genomic DNA was extracted using the Qiagen DNAeasy Blood&Tissue kit following the standard protocol. A fragment of the mitochondrial cytochrome *c* oxidase subunit I (COI) gene was amplified via PCR (Saiki et al. 1988) using the Nancy (Simon et al. 1994) and LCO (Folmer et al. 1994) primer pair following previously published protocols (Wesener et al. 2010). Both strands were sequenced by Macrogen (Macrogen Europe Laboratory, Amsterdam, The Netherlands), following the Sanger sequencing method (Sanger et al. 1977). Sequencing reads were assembled and aligned by hand with Bioedit 7.1.3 (Hall 1999) and confirmed with BLAST searches (Altschul et al. 1997). Sequences were uploaded to GenBank (Accession numbers: KP205571 to KP205557).

**Figure 2.**
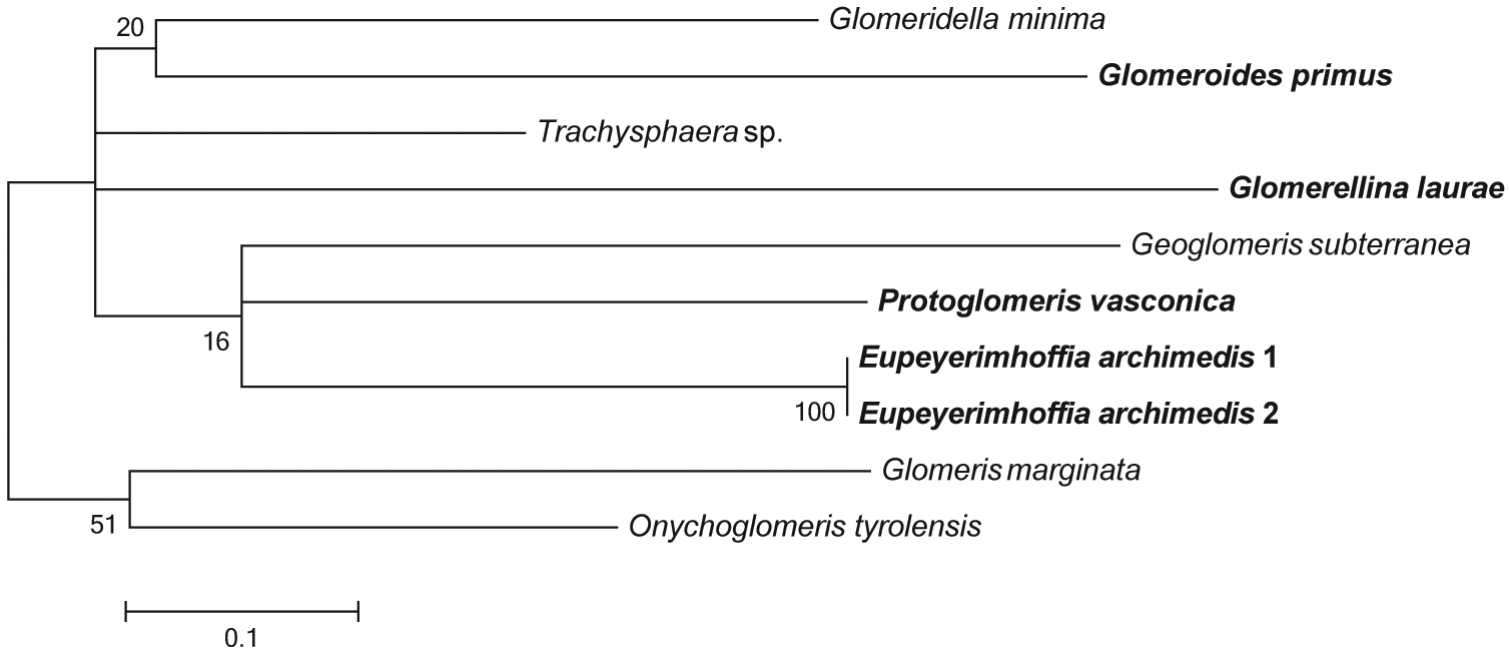
Maximum likelihood bootstrap consensus tree. Members of the family Protoglomeridae are marked in bold.

Mean pairwise distances between terminals (transformed into percentages) were determined using MEGA5.2 (Tamura et al. 2011). To better illustrate relationships between genera, a maximum likelihood phylogenetic analysis was conducted in MEGA5.2 (Tamura et al. 2011). The implemented ModelTest selected the HKY+G+I model (Hasegawa et al. 1985) as best-fitting (BIC = 5783.1, -lnL = -2791.2354, freqA = 0.2647, freqC = 0.1366, freqG = 0.2014, freqT = 0.3972, gamma shape = 0.3364). The bootstrap consensus tree (Fig. 2), inferred from 1000 replicates (Felsenstein 1985), is used to represent the evolutionary history of the analyzed taxa. All positions containing gaps and missing data were eliminated. There were a total of 668 positions in the final dataset.

While the genetic marker used does not allow a study of the phylogeny of the group, first insights into the separation of the genera are provided.

**Table 2.** Pair-wise uncorrected p-distances (%) of the COI-fragment.

#	Species	1	2	3	4	5	6	7	8	9
1	*Glomeris marginata*									
2	*Glomeridella minima*	16.0								
3	*Geoglomeris subterranea*	17.4	15.6							
4	*Trachysphaera* sp.	15.0	13.2	15.6						
5	*Glomeroides primus*	16.3	14.2	16.4	15.0					
6	*Onychoglomeris tyrolensis*	14.3	13.2	16.8	13.2	15.3				
7	*Protoglomeris vasconica*	14.8	12.0	15.3	13.5	15.0	13.2			
8	*Glomerellina laurae*	18.8	16.0	18.3	15.3	16.7	16.3	15.8		
9	*Eupeyerimhoffia archimedis* 1	16.1	15.2	15.0	13.1	16.5	15.0	13.2	16.8	
10	*Eupeyerimhoffia archimedis* 2	16.2	15.3	15.0	13.2	16.7	15.2	13.4	16.8	0.2

## Results

### Distance analysis

The uncorrected pairwise distances between genera included in the present study are relatively high. The genetic distances are not lower between species within the same family than between species of different families. The distances range from 18.8% between *Glomerellina laurae* (Protoglomeridae) and *Glomeris marginata* (Glomeridae) to 12.0% between *Protoglomeris vasconica* (Protoglomeridae) and *Glomeridella minima* (Glomeridellidae). The two *Eupeyerimhoffia archimedis* samples show a 0.2% sequence divergence, but also show both the highest (16.8%: *Glomerellina laurae*) and lowest distance (13.2 and 13.4%: *Protoglomeris vasconica*) to other species within the family.

### Tree description

The maximum likelihood tree receives little to no support, most nodes remain unresolved and all taxa are separated by long branches (Fig. 2). The family Protoglomeridae (P) could not be recovered. All members of the family are recovered within a major polytomy together with species from both Glomeridellidae and Glomeridae (Fig. 2). Within the polytomy *Glomeroides primus* (Protoglomeridae) groups together with *Glomeridella minima* (Glomeridellidae). *Glomerellina laurae* (P) does not cluster with any species within the polytomy and rests on the longest branch within the tree. *Protoglomeris vasconica* (P) and *Eupeyerimhoffia archimedis* (P) are recovered in a polytomy together with *Geoglomeris subterranea* (Glomeridae). Only the subfamily Glomerinae (*Glomeris* & *Onychoglomeris*) could be resolved as monophyletic (Fig. 2) as the sister group to the polytomy, though with weak support.

### Family Protoglomeridae Brölemann, 1913

**Diagnosis.** Simple telopods with four podomeres distal to syncoxite, forming pincers. Telopoditomeres 1–3 lacking trichosteles. Telopoditomere 2 with a non-membranous immovable finger located almost parallel to telopoditomere 3. Here we follow the typological system of Mauriès (2005), despite the fact that no phylogenetic analysis has been undertaken to characterize the families in the order.

#### Eupeyerimhoffia

Taxon classificationAnimaliaGlomeridaProtoglomeridae

Brölemann, 1913

Eupeyerimhoffia Brölemann, 1913: 166–174 (first description); Jeekel 1971: 13 (note); Strasser 1976: 581–583 (synonymization *Trinacriomeris*); Hoffann 1980: 67 (list); Foddai et al. 1995: 11 (list); Shelley et al. 2000: 11 (list); Mauriès 2005: 242 (classification); Kime and Enghoff 2011: 39 (atlas).
Trinacriomeris Strasser, 1965: 10–14. syn.

##### Diagnosis.

Tergite 11 fused to anal shield. Telopod simple with four podomeres. Process of telopoditomere 2 of telopod short and stout. Male leg-pair 17 with four podomeres. Coxa of male leg-pair 18 not fused to syncoxite. Lateral palpi of gnathochilarium as large as inner palpi. One of the largest Glomerida, 18–22 mm long. Mandible with large condylus. Members might be mistaken in habitus, size and color with the species of the genus *Onychoglomeris* Verhoeff, 1909, whose species differ greatly in the telopods and many other characteristics.

##### Type species.

*Eupeyerimhoffia algerina* Brölemann, 1913 from Algeria

##### Other species included.

*Eupeyerimhoffia archimedis* (Strasser, 1965) from southern Sicily.

#### Eupeyerimhoffia archimedis

Taxon classificationAnimaliaGlomeridaProtoglomeridae

(Strasser, 1965)

Trinacriomeris archimedis Strasser 1965: 10–14 (first description);
Trinacriomeris archimedis Strasser 1970: 153 (list);
Trinacriomeris archimedis Strasser 1976: 581–583 (synonymization *Trinacriomeris*);
Eupeyerimhoffia archimedis Foddai et al. 1995: 11 (list);
Trinacriomeris archimedis Shelley et al. 2000: 11 (list).

##### Material examined.

1 F, **MHNG**, lectotype (designated herewith), labeled paratype, in 70% ethanol, Italie (Sicile): Siracuse: Avola pr. Siracuse. 1 F, **MHNG 3460**, dried and mounted, Italie (Sicile): Siracuse: Avola pr. Siracuse; 1 F, **ZFMK MYR01879**, 1 M, **ZFMK MYR01875**, Italy, Sicily at type locality, south of Ferla, 37.1151333°N, 014.9403667°E, coll. J.P. Oeyen & P. Erkeling, 10.vii.2013; 1 F, **ZFMK MYR 1965**, Italy, Sicily, Province Syracuse, East of Palazzolo Acreide, Ravine, deciduous forest, 37.0997667°N, 015.0232000°E, coll. J.P. Oeyen & P. Erkeling, 13.vii.2013.

##### Comment.

A second female type specimen from Ferla, Sicily was, according to the first description, stored at the University of Catania, Institute of Zoology, Italy.

##### Re-diagnosis.

Can easily be distinguished from the other Sicilian Glomerida species by size and color. It is the largest and only light brown species on the island. It can be distinguished from its congener *Eupeyerimhoffia algerina* in having: (1) Single continuous anterior stria on collum, posterior stria divided in lateral parts; (2) thoracic shield with single continuous stria reaching the lateral lobes on both sides.

##### Description.

**General coloration** (living specimen) light brown, almost copper. Collum, head, antennae, posterior margin and lateral speckled fields of tergites lighter, almost golden cream color (Fig. 1A, B).

**Figure 3.**
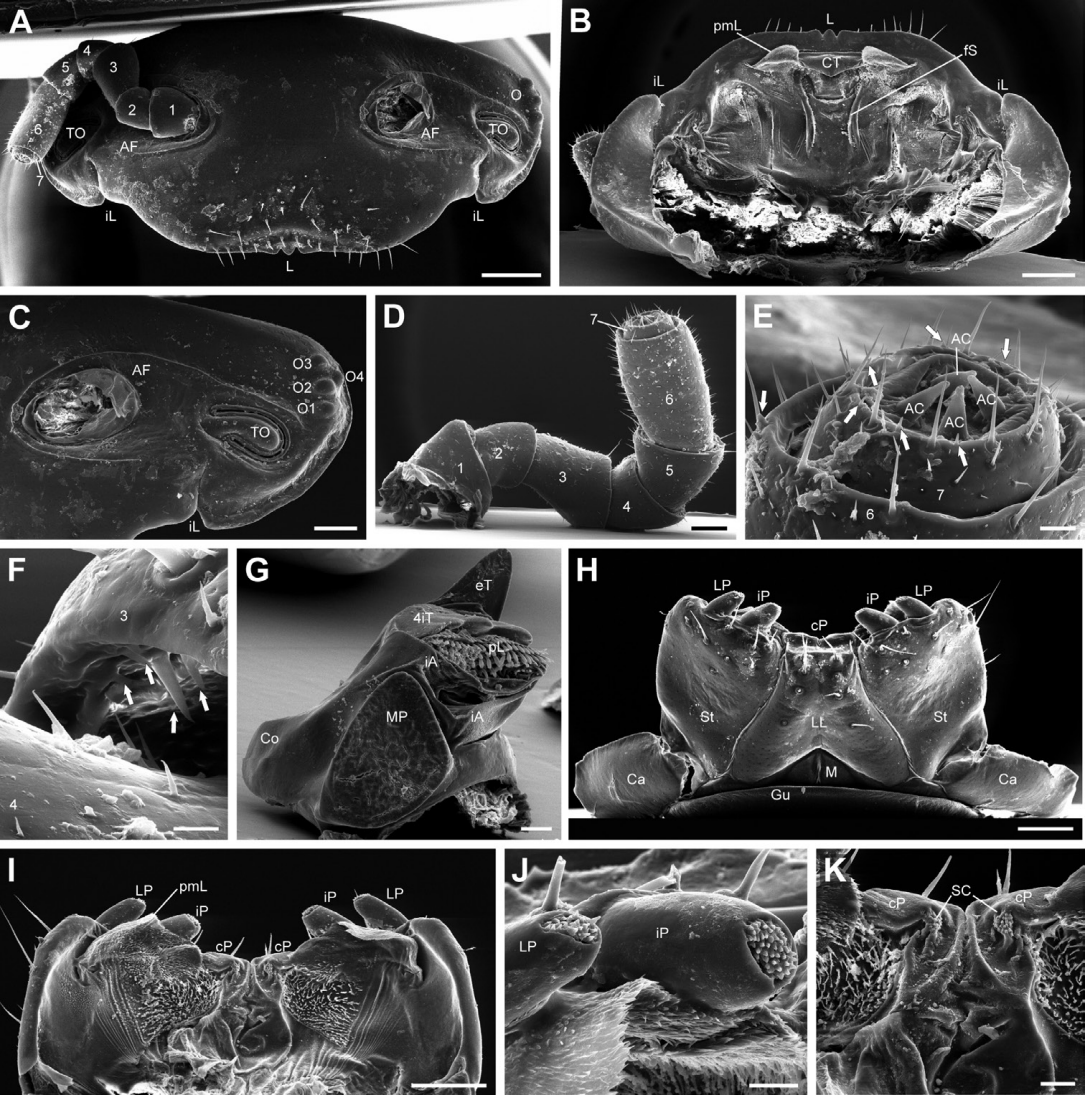
*Eupeyerimhoffia archimedis* (Strasser, 1965) male, SEM. **A** Head, frontal view **B** Head, ventral view **C** Head, detail of lateral area **D** Antenna, posterior view**E** Antenna, antennomere 6 and 7 **F** Antenna, apical edge of antennomere 3 **G** Mandible, mesal view **H** Gnathochilarium, ventral view **I** Endochilarium, dorsal view **J** Gnathochilarium, lateral and inner palpi, dorsal view **K** Endochilarium, detail of median area, dorsal view. Abbreviations: 1-7 = Antennal segments number 1-7; 4iT = 4-combed inner tooth; AC = Apical cone; AF = Antennal fossa; Ca = Cardine; Co = Condylus; cP = Central pads; CT = Central tooth; eT = External tooth; fS = fringed seam; Gu = Gula; iA = Intermediate area; iL = Incisura lateralis; iP = Inner palpi; L = Labrum; LL = Lamella linguales; LP = Lateral palpi; M = Mentum; MP = Molar plate; O = Ocellaria; O1-O4 = Ocelli 1-4; pL = Pectinate lamellae; pmL = paramedian lobe; TO = Organ of Tömösváry; SC = Sensory clusters; St = Stipites. Arrows mark *sensilla basiconica*. Scale bar: 400 μm (**A, B**); 200 μm (**C**); 150 μm (**D**); 25 μm (**E**); 10 μm (**F**); 100 μm (**G**); 250 μm (**H, I**); 40 μm (**J**); 50 μm (**K**).

**Head** sparsely covered with minute setae, >10 supralabral setae (Fig. 3A, C). Incisura lateralis (IL) directed slightly laterally, not reaching height of organ of Tömösváry (TO) or antennal basis (Fig. 3A–C). Lateral marginal bulge thickest at IL, decreasing gradually dorsally until terminating at height of dorsal-most ocellus (Fig. 3C). A furrow running laterally between ventral-most ocellus and TO, circumventing antennal fossa and terminating at height of IL (Fig. 3C).

**Labrum** wide, with 19 marginal setae (Fig. 3A, B). Central labral tooth projecting beyond lateral margin.

**Epipharynx** with pronounced central tooth and two lateral membranous lobes, covered densely in cuticular scales (Fig. 3B). Incisura lateralis clearly visible, reaching margin of head capsule. Two paramedian fringed seams stretching from central tooth posteriorly towards hypopharynx.

**Ocellaria** black, 3+1 convex lenses (Fig. 3C).

**Antennae** with four apical cones (Fig. 3E). Antennomere 3 approximately as long as 1 and 2 combined (Fig. 3D). Antennomere 6 approximately 1.9 times longer than wide. Antennomeres 1–5 only sparely setose, 6th more densely setose. Multiple sensilla basiconica on proximal apical edge of antennomere 3 (Fig. 3F) as well as at apical edge of antennomere 7 (Fig. 3E).

**Organ of Tömösváry** recessed, elongate, curved ventrally (Fig. 3C). 1.9 times longer than wide. Bulging cone and slit margins smooth. Cone narrower at midpoint. No internal structures visible in SEM.

**Gnathochilarium** ventrally with 8 large setae on lamella linguales, 12 large setae on each stipites (Fig. 3H). Remaining ventral surface glabrous. Cardines large. Inner palpi slightly larger than lateral palpi (Fig. 3H–J). Inner palpi with >40 sensory cones standing in single field (Fig. 3J). Lateral palpi also with field of >20 sensory cones (Fig. 3J).

**Endochilarium** with large anterior membranous paramedian lobes (pmL), densely covered with cuticular scales (Fig. 3I). Fields of long setae posterior to membranous lobes. Central pads with single cluster of sensilla directed towards median furrow (Fig. 3I, K).

**Mandible** with single large outer tooth and four-combed inner tooth (Fig. 3G). Proximal comb of inner tooth slightly ovoid. Six rows of pectinate lamellae. Lateral areas of intermediate area covered with small cuticular scales, central part smooth with possible pore. Molar plate almost triangular, marginal bulge at anterior edge, no anterior depression and posterior tip slightly curved towards mandibular basis. Condylus pronounced (Fig. 3G).

**Figure 4.**
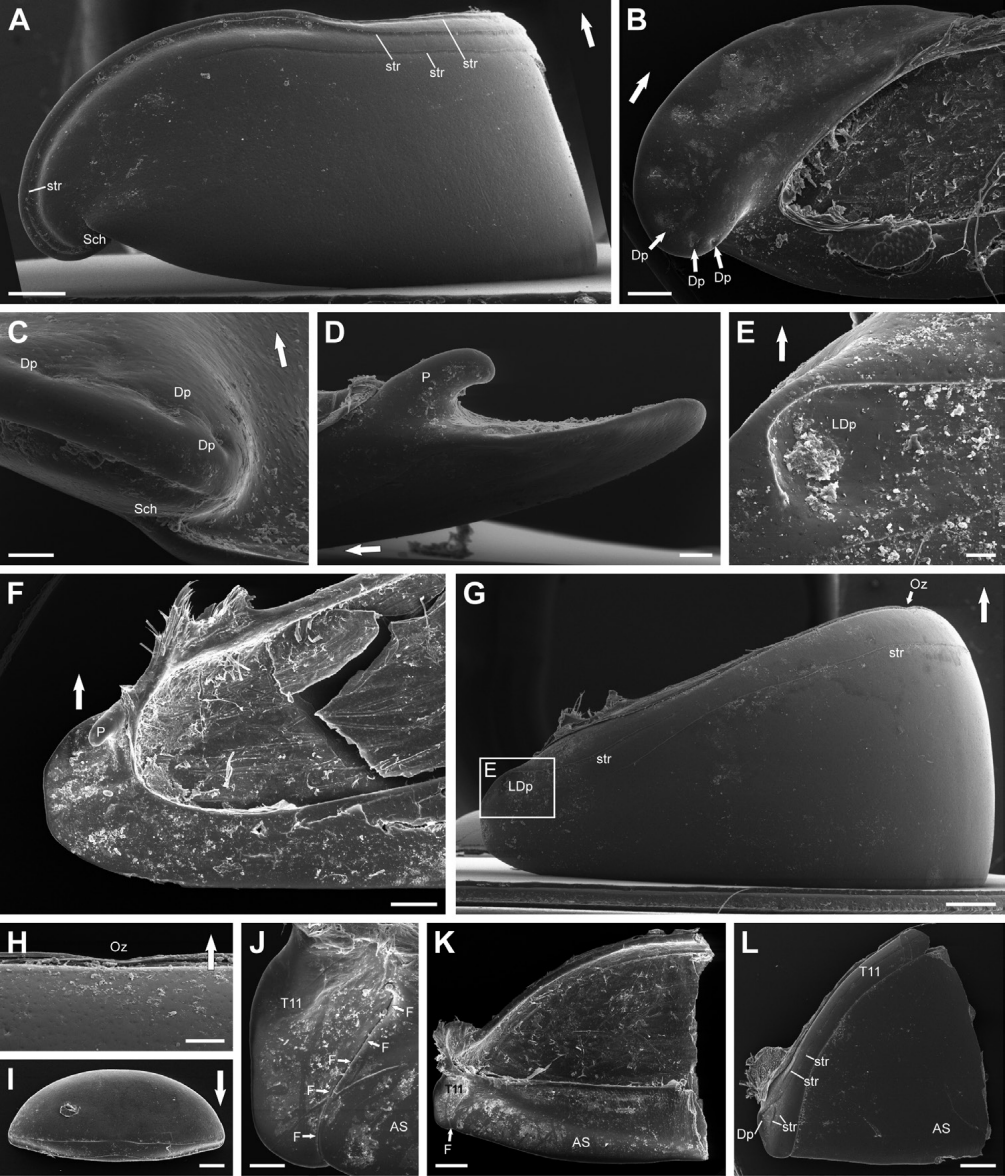
*Eupeyerimhoffia archimedis* (Strasser, 1965) male, SEM. **A** Thoracic shield, dorso-lateral view **B** Thoracic shield, meso-lateral **C** Thoracic shield, schism detail, ventro-lateral view **D** Tergite, detail of peg, antero-lateral view **E** Tergite, detail of depression, lateral view **F** Tergite, ventral view **G** Tergite, dorso-lateral view **H** Tergite, ozopore, dorsal view **I** Collum, dorsal view **J** Tergite 11 and anal shield, detail of furrow, ventro-lateral view **K** Tergite 11 and anal shield, left side, anterior view **L** Tergite 11 and anal shield, right side, posterior view. Abbreviations: AS = Anal shield; Dp = Depression; F = Furrow; Oz = Ozopore; LDp = Lateral depression; P = Peg; Sch = Schisma; stri = striae; T11 = Tergite 11. Arrows point anteriorly. Scale bar: 400 μm (**A, H, I, K, L**); 300 μm (**B, F**); 100 μm (**C, D, J**); 50 μm (**E**); 500 μm (**G**).

**Collum** with one continuous anterior and two posterior lateral striae (Fig. 4I). Uniformly covered with minute setae, recessed into small pits.

**Thoracic shield** with very small schism (Fig. 4A). 3 median striae. Marginal furrow widest laterally, narrowing medially. Uniformly covered with minute recessed setae. Ventral area of lobe with seven anterior marginal depressions on lobe and a single depression at the posterior margin (Fig. 3B, C; see below for function of depressions).

**Tergites 3–10** covered with minute recessed setae, with single complete transverse anterior stria and short lateral striae anteriorly circumventing a depression (Fig. 4E, G). Lateral edges not projecting posteriorly. Stout pegs on ventral areas projecting posteroventrally from lateral most part of anterior edge (Fig. 4D, F).

**Ozopore** simple, neither with special sutures nor other structures (Fig. 4H).

**Tergite 11 and anal shield** completely fused but both dorsally and ventrally distinguishable by a pronounced furrow (Fig. 4J–L). Tergite 11 with 3 short lateral striae and a single stria circumventing a lateral depression and stretching across whole tergite (Fig. 4L). Tergite 11 and anal shield dorsally evenly covered with minute setae, with neither any special notches nor structures.

**Pleurites** evenly covered with small setae, bulge at anterior edge widest medially narrowing towards proximal edge. Pleurite 1.2 times wider than long.

**Stigmatic plates** reaching around coxa on both anterior and posterior sides. 1.5 times wider than long, almost pentagonal in shape. Plate with regular margin, lacking any projections. Spiracle inconspicuous, protected by small knob.

**Figure 5.**
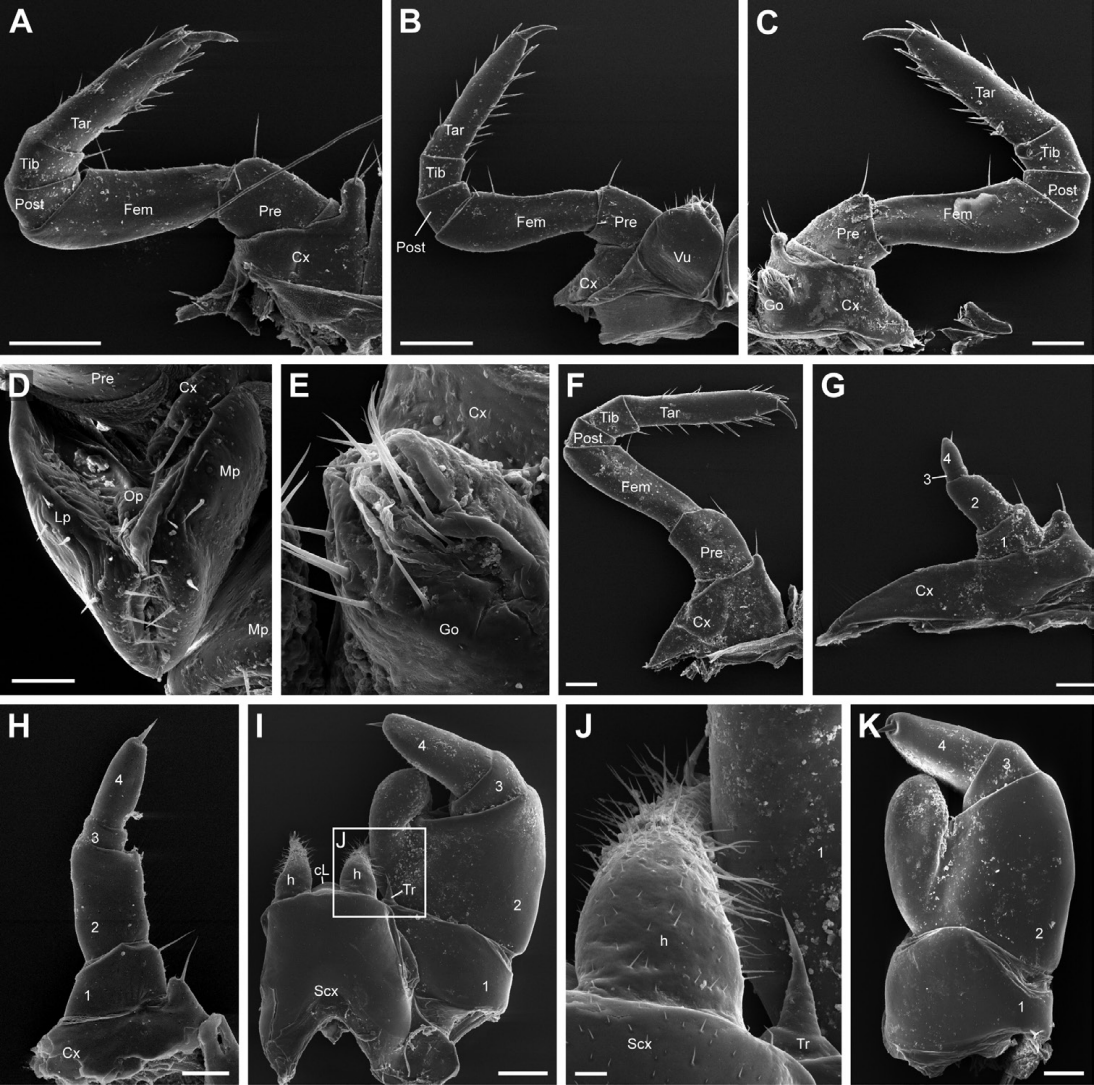
*Eupeyerimhoffia archimedis* (Strasser, 1965) male and female, SEM. **A** Leg-pair 1, male, right side, posterior view **B** Leg-pair 2, female, right side, posterior view **C** Leg-pair 2, male, left side, posterior view **D** Leg-pair 2, female, right vulva, ventral view **E** Leg-pair 2, male gonopore, posterior view **F** Leg-pair 9, male, right side, posterior view **G** Leg-pair 17, left side, anterior view **H** Leg-pair 18, right side, posterior view **I** Telopod with syncoxite, anterior view **J** Telopod, inner horn of syncoxite **K** Telopod, posterior view. Abbreviations: Cx = Coxa; Pre = Prefemur; Fem = Femur; Post = Postfemur; Tib = Tibia; Tar = Tarsus; Vu = Vulva; Go = Gonopore; Mp = Median plate; Lp = Lateral plate; Op = Operculum; 1-4 = Podomere 1-4; cL = Central lobe; h = Inner horn; Scx = Syncoxite; Tr = Trichostele. Scale bar: 400 μm (**A, B**); 250 μm (**C**); 100 μm (**D, H**); 25 μm (**E**); 200 μm (**F, I**); 150 μm (**G, K**); 50 μm (**J**).

**Midbody legs** sparsely covered with minute setae (Fig. 5F). Coxa almost triangular, much wider at base than apically. Coxa mesally elongated to process carrying a single spine. Two coxal furrows originating at center of coxal basis, one stretches apically around coxa, the second terminates after 2/3 of coxal height in a meso-apical direction. Tibia, pre- and postfemur with a single mesal spine, femur with two. Apical margin of prefemur with a single small apical protrusion. Femur almost 3 times longer than wide. Tarsus with no apical, 11 dorsal and 8–11 ventral spines. Tarsus 4.5 times longer than wide. Claw elongated.

**Male sexual characters.**

**Male tergite 11 and anal shield** do not show any special structures (Fig. 4J–L). See further and more detailed descriptions above.

**Male first leg-pair** sparsely covered with minute setae (Fig. 5A). Coxa not widened at basis, but mesally elongated to a process carrying two spines. Postfemur and tibia each with single mesal apical spine, prefemur and femur with two. Apical margin of prefemur with a single small protrusion. Tarsus with 7–10 dorsal and 8 ventral spines. Claw elongated but stout at basis. Tarsus almost 4 times longer than wide.

**Male second leg-pair** similar to midbody legs, but with a bulbous medial coxal protrusion carrying two spines (Fig. 5C), similar to leg 1. Tarsus approximately 3.8 times longer than wide.

**Male gonopore** clam-shaped and mesally protruding from posterior side of coxa 2 (Fig. 5E). Single elongate membranous opening surrounded by 9 or 10 apical and 4 basal setae. No division into separate plates.

**Male leg 17** reduced with 4 podomeres (Fig. 5G). Coxa with small medial process bearing a subapical spine and a wide but narrow, almost triangular, coxal lobe. Apical edge of coxa with small protrusion. Podomere 1 with mesal spine. Second podomere approximately 1.8 times wider than podomere 3. Podomere 3 very short and inconspicuous. Podomere 4 with subapical spine. Complete leg sparsely covered with minute setae.

**Male leg 18** reduced, but to a lesser extent than leg-pair 17 (Fig. 5H). Coxa slightly damaged during dissection, but apparently without widened coxal lobe and not fused to syncoxite. Small mesal coxal process with single subapical spine. Single, well-developed medial spine on podomere 1. Apical edge of podomere 1 with apical protrusion. Podomere 2 approximately 1.5 times as wide as podomere 3. Podomere 3 very short, no spines and with very inconspicuous borders to podomere 4. Podomere 4 with apical spine.

**Telopod** (male leg 19) stout, syncoxite likewise (Fig. 5I–K). Syncoxal lobe small and rounded. Inner horns of syncoxite with numerous hairs of varying length and well-developed subapical spine, which is curved almost 90° (Fig. 5J). Telopoditomere 1 with mesal, highly reduced trichostele (Fig. 5I, J). Telopoditomere 2 mesally elongated into large bulbous process (immovable finger) with knobbed proximal surface. Telopoditomere 3 short, approximately 2 times wider than long, devoid of any peculiarities. Telopoditomere 4 with medial field of knobs juxtaposed to process of telopoditomere 2, and a large posteriorly oriented spine. Telopoditomere 4 forms chela (pincer) against medial process of second telopoditomere.

**Female sexual characters.**

**Female second leg-pair** similar to midbody legs, but coxa with two spines on separate medial protrusions which are fused basally (Fig. 5B). Tarsus 4 times longer than wide.

**Female vulva** large, attached to posterior side of coxa via membranes (Fig. 5D). Operculum recessed between vulva plates. Posterior end of operculum narrower than anterior one, with two spines. Vulva with ventrally symmetrical mesal and lateral plates, carrying altogether 7 or 8 spines. Lateral plate overlaps mesal one apically on posterior side, but both are fused together at their base via a membranous connection.

##### Intraspecific variation.

Not enough samples present to describe morphological variation. The populations from Ferla and Palazzolo Acreide have two different haplotypes, differing at one base pair position.

##### Volvation.

As described by Strasser (1965), the genus has a volvation strategy which differs from what is known from most other Glomerida. When rolling up into a ball the ventral ends of the tergites are not inserted in the schisma of the thoracic shield, unlike in *Glomeris*, but rest on top of it (Fig. 1A). The pegs on the ventral side of the tergites (Fig. 4D, F) rest within the depressions on the ventral side of the thoracic shield (Fig. 4B, C).

##### Habitat.

All of the samples were collected during the day in deciduous forests. Specimens were mainly found in the leaf litter or under small stones. Curiously, some were also found in close proximity to ant nests (Hymenoptera) and under moss growing directly on an exposed rocky surface.

## Discussion

### Problems during the morphological analysis:

The described position of the vulva operculum might be an artifact, as the structural integrity of membranous structures was not preserved by critical point drying. This should be considered for future studies of glomerid vulvae.

The sampling within the present study did not allow for a description of the morphological variation within the species. However, the 0.2% sequence divergence between the two reported localities shows that there is variability within the species, with at least two haplotypes present on the island.

### Unique morphological characters of *Eupeyerimhoffia*

*Eupeyerimhoffia archimedis* shows several interesting characters. The mandible with a large condylus and flat molar plate lacking a groove (Fig. 3G) is very atypical of Glomerida. Glomerida are generally described as lacking a condylus and always possessing a molar plate with a distinct deep groove (Köhler and Alberti 1990). Furthermore, contrary to previous descriptions, the Protoglomeridae-like telopods possess a trichostele on the first podomere, which represents another special character of the species, if not of the genus. The presence of this trichostele violates the diagnosis of the family as proposed by Mauriès (2005).

### Volvation strategy

The volvation strategy of *Eupeyerimhoffia* is another striking and possibly unique feature of the genus inside the order Glomerida. Similar pegs on the tergites have been reported for members of the genera *Epiromeris* (Thaler and Knoflach-Thaler 1998) and *Trachysphaera* (Strasser, 1965). These do not, however, possess the herein described thoracic shield lobe with a reduced schisma in combination with ventral depressions (Fig. 4A–C). Both modifications allow *Eupeyerimhoffia* a unique method of rolling into a ball. To understand this phenomenon, further inquiries into the origin and diversification of glomerid volvation are necessary and jointly represent a very interesting future research topic on its own.

### Relationships of the four genera of the Protoglomeridae and impact on Glomerida phylogeny

As stated above, the COI fragment is not well suited to study the group’s phylogeny. Therefore it is not surprising that the COI tree lacks resolution and receives little statistical support. Nonetheless, together with the distance analysis, it is sufficient to observe that the members of the family Protoglomeridae are not each other’s closest relatives (e.g. *Glomeroides primus* grouping with *Glomeridella minima* from the separate suborder Glomeridelloidea) and possibly that the family does not constitute a monophyletic unit. Similar results have also been reported by Wesener (2012) in a study that did not include all members of the family. This supports the notion that characters based mainly on the telopods are not sufficient to infer relationships within the order Glomerida. This is especially true when considering the close relationship between *Eupeyerimhoffia archimedis* and *Protoglomeris vasconica*, despite the fact that *Eupeyerimhoffia archimedis* does not conform to the diagnosis of the family. Therefore, a phylogenetic analysis based on a much broader dataset, including further molecular markers and morphological characters, is required to illuminate the evolutionary history of the pill millipedes.

## Supplementary Material

XML Treatment for Eupeyerimhoffia

XML Treatment for Eupeyerimhoffia archimedis
